# Study of negative pressure wound therapy as an adjunct treatment for acute burns in children (SONATA in C): protocol for a randomised controlled trial

**DOI:** 10.1186/s13063-019-3223-9

**Published:** 2019-02-13

**Authors:** Cody C. Frear, Bronwyn Griffin, Leila Cuttle, Steven M. McPhail, Roy Kimble

**Affiliations:** 1 Centre for Children’s Burns and Trauma Research, Level 7, Children’s Health Research Center, The University of Queensland, 62 Graham St., South Brisbane, QLD 4101 Australia; 2 The Pegg Leditschke Children’s Burns Centre, Queensland Children’s Hospital, Lvl. 5, 501 Stanley St., South Brisbane, QLD 4101 Australia; 3 The University of Queensland Faculty of Medicine, 288 Herston Rd., Brisbane, QLD 4006 Australia; 4 Institute of Health and Biomedical Innovation, Centre for Children’s Burns and Trauma Research, Lvl 8, Children’s Health Research Centre, Queensland University of Technology, South Brisbane, QLD 4101 Australia; 5 Centre for Functioning and Health Research, Metro South Health, Queensland Health, Brisbane, QLD 4102 Australia; 6 School of Public Health & Social Work and Institute of Health and Biomedical Innovation, Queensland University of Technology, Brisbane, QLD 4059 Australia

**Keywords:** Paediatric, Burns, Negative-pressure Wound Therapy, Re-epithelialisation, Pain, Burn wound progression, Itch, Randomised controlled trial

## Abstract

**Background:**

Although negative pressure wound therapy (NPWT) is widely used in the management of several wound types, its efficacy as a primary therapy for acute burns has not yet been adequately investigated, with research in the paediatric population particularly lacking. There is limited evidence, however, that NPWT might benefit children with burns, amongst whom scar formation, wound progression and pain continue to present major management challenges. The purpose of this trial is to determine whether NPWT in conjunction with standard therapy accelerates healing, reduces wound progression and decreases pain more effectively than standard treatment alone.

**Methods/design:**

A total of 104 children will be recruited for this trial. To be eligible, candidates must be under 17 years of age and present to the participating children’s hospital within 7 days of their injury with a thermal burn covering <5% of their total body surface area. Facial and trivial burns will be excluded. Following a randomised controlled parallel design, participants will be allocated to either an active control or intervention group. The former will receive standard therapy consisting of Acticoat™ and Mepitel™. The intervention arm will be treated with silver-impregnated dressings in addition to NPWT via the RENASYS TOUCH™ vacuum pump. Participants’ dressings will be changed every 3 to 5 days until their wounds are fully re-epithelialised. Time to re-epithelialisation will be studied as the primary outcome. Secondary outcomes will include pain, pruritus, wound progression, health-care-resource use (and costs), ease of management, treatment satisfaction and adverse events. Wound fluid collected during NPWT will also be analysed to generate a proteomic profile of the burn microenvironment.

**Discussion:**

The study will be the first randomised controlled trial to explore the clinical effects of NPWT on paediatric burns, with the aim of determining whether the therapy warrants implementation as an adjunct to standard burns management.

**Trial registration:**

Australian New Zealand Clinical Trials Registry, ACTRN12618000256279. Registered on 16 February 2018.

**Electronic supplementary material:**

The online version of this article (10.1186/s13063-019-3223-9) contains supplementary material, which is available to authorized users.

## Background

The acute burn wound is a complex, dynamic injury that presents a range of challenges for patients and clinicians alike. It has been described as “the ultimate inflammatory injury” [1] and “amongst … the most devastating afflictions on the human body” [2]. Thanks to improvements in the management of severe burns, as well as public health and infrastructural advances [3], mortality due to burns has dropped substantially in the past half century [4]. Current American and Australian in-hospital death rates stand at 3% and 2%, respectively [5, 6]. Yet burns remain one of the most common types of trauma [4, 7], particularly in children, whose developing psychomotor skills, poor spatial awareness and exploratory behaviour place them at increased risk of injury [8].

In the US and Australia, children constitute more than one third of all burns-related hospital admissions [5, 6]. Admissions, however, capture only a small percentage of the true magnitude of burn injuries in children, as between 87% and 93% of paediatric burns are treated exclusively as outpatients [9, 10]. This is attributable, in large part, to the exceeding rarity of severe life-threatening burns in high-income countries. At one of Australia’s largest tertiary paediatric burns centres, over 62% of patients present with wounds covering less than 1% of their total body surface area (TBSA), and more than 90% of all burns are thermal in origin (i.e., scalds, contact burns, flame burns or radiant heat burns) [9]. The predominant focus in modern paediatric burns care, therefore, is no longer the treatment of severe burns and their life-threatening systemic effects, but rather the improvement of functional and cosmetic outcomes in small-to-medium-sized thermal wounds.

In recent decades, the acute management of such injuries has been advanced by innovations such as early excision and grafting [11–14], skin substitute technologies [15–18] and silver-impregnated dressings [19]. Nevertheless, complications including scar formation [20], contractures [21] and pain [22] still commonly affect children who have sustained burns. In particular, hypertrophic scarring, which is defined as the formation of raised scars within the boundaries of a wound, occurs in high proportions of burns patients (with a reported prevalence ranging from 32% to 72%) [23]. For children especially, the long-term physical and psychological consequences of these scars can be devastating [24]. In addition to their effects on appearance, self-esteem and social acceptance [25–28], they can lead to contractures that impair the range of motion and function as a child grows, necessitating recurrent surgical operations to release the scar tissue [21].

The facilitation of wound closure represents one of the central aims of acute burns treatment. Prompt wound closure is essential for the prevention of scarring, as there has long been a well-understood relationship between time to re-epithelialisation and scar formation. A widely held model that was first described by Deitch et al*.* [29] and later supported by others [30, 31] identified the 3-week mark as the critical time point in the healing process with regard to scarring. Burns that fully re-epithelialise before this point have a low risk of developing hypertrophic scars (and an especially low risk if healed within 2 weeks), whilst those requiring greater than 3 weeks are highly likely to scar. Although more recent research has somewhat challenged this doctrine by finding lower rates of scarring amongst the latter group than previously reported [32]—a change some authors attribute anecdotally to the increasing use of silver-impregnated dressings [31]—burns clinicians still overwhelmingly agree that prolonged healing is a major predictor for scar formation and regard any methods aimed at accelerating re-epithelisation as worthy of consideration.

Time to re-epithelialisation is closely tied to burn depth, of which there are four broad classifications [33]. In superficial burns, only the epidermal layer of the skin is affected. Superficial partial-thickness and deep dermal partial-thickness burns extend to the papillary and reticular layers of the dermis, respectively. Burns involving the entirety of both the epidermis and dermis are categorised as full- thickness. Superficial partial-thickness burns tend to heal spontaneously within 2 weeks, whilst deep dermal partial-thickness burns exhibit more prolonged healing, typically taking 3 to 5 weeks to fully re-epithelialise. Spontaneous wound closure is impossible for full-thickness burns, which heal exclusively at the margins by scarring. The delay or inhibition of the healing process in deeper burns stems in large part from the destruction of adnexal structures in the reticular dermis. These structures, which include hair follicles, sebaceous glands and sweat glands, serve as reservoirs of keratinocytes, which can repopulate zones of cellular damage [34]. Since deeper burns are associated with significant scarring [29], they are almost always treated with debridement and grafting [35].

Burns are dynamic injuries that are vulnerable to significant changes in size, depth and severity due to a process known as burn wound conversion. This phenomenon is best understood by reviewing Jackson’s burn wound model [36], which holds that thermal injuries are characterised locally by three concentric zones. The core zone of coagulation contains irreversibly destroyed necrotic tissue. By contrast, the outermost zone of hyperaemia is marked by increased blood flow and inflammation but will invariably recover. It is within the intermediate zone between these two, the zone of stasis, that appropriate management is most vital. Tissue here remains at least temporarily viable, but if the burn is not adequately treated, may undergo progressive cellular damage and death that will result in recruitment into the zone of coagulation. A complex interplay of pathological mechanisms underlies this process.

The initial thermal insult results in a prolonged inflammatory reaction characterised by the accumulation of neutrophils [37, 38]. In addition to releasing harmful cytokines and reactive oxygen species associated with collagen denaturation, keratinocyte apoptosis and DNA damage [37, 39], these neutrophils adhere to the endothelial lining of local blood vessels [40], leading to vascular plugging. Leukocyte adherence, in combination with increased vascular permeability and reduced interstitial hydrostatic pressure [41, 42], also contributes to oedema formation [43], which itself significantly impairs perfusion to the burn wound. Local circulation is further compromised by microvessel thrombosis arising from a peak in hypercoagulability that occurs 2 to 3 hours following injury [44]. Finally, oxidative stress, linked with over-activity of xanthine oxidase and NADPH oxidase, damages cellular proteins, nucleic acids and lipids central to the healing process [45, 46]. Subsequent expansion of the depth and surface area of a burn can continue for up to 5 days post-injury [47]. Adequate first aid and the application of appropriate dressings may alleviate some of these factors [48], but no techniques have yet been found that definitively minimise burn wound progression [49].

Even more prevalent and difficult to prevent than scarring, pain persists as one of the greatest unmet challenges in burns management [50]. Despite the development of sophisticated analgesic protocols, unrelieved pain continues to be reported at high rates amongst burns patients, often ranking as their most frequent complaint [51]. The risks of undertreated pain are enormous: in addition to causing intense momentary suffering, anxiety and distress, it can contribute to delayed healing [52] and the development of long-term sensory disturbances [53–55], chronic pain [50] and debilitating psychological issues [56, 57]. Notably, the highest levels of pain intensity and undertreatment are associated with procedural pain resulting from dressing changes and other interventions [58–60].

A number of alternative or adjunct therapies have been proposed to help facilitate the healing process, decrease burn wound progression and minimise pain [47]. One promising approach is negative pressure wound therapy (NPWT), a technique that involves the use of a vacuum device to establish subatmospheric pressures through occlusive dressings over a wound site [61, 62]. Also known as vacuum-assisted closure, topical negative pressure, subatmospheric pressure and reticulated open cell foam therapy [63], NPWT shares the same basic principles as other medical therapies that have been in use for well over a century [64–66], but the current form of the technology, characterised by the maintenance of an evenly distributed vacuum through foam or gauze, first became commercially available in 1995 [67].

Since then, NPWT has become widely accepted in the world of wound management, particularly in the context of diabetic ulcers [68], open abdomens [69, 70], sternal wounds [71], open fractures [72] and haemangiomas [73]. Although the exact mechanism of action of NPWT is not yet fully understood, it is known to exert several effects on the local tissue environment. Among the most notable is a rise in extracellular pressure [74, 75]. This seemingly paradoxical trend is likely a result of tissue macrodeformation: as air is evacuated from the NPWT foam or gauze, the volume of the packing material decreases, consequently compressing the adjacent tissue. NPWT is believed to aid the healing process specifically by inducing wound contraction [76], generating microdeformational changes that stimulate cellular proliferation and neovascularisation [77–79], evacuating oedema fluid that adversely affects the local microvasculature, [80, 81] extracting toxins and bacteria [62, 82, 83], and preventing desiccation of the wound environment [63, 84].

NPWT has been employed at various stages in the management of burns, with the vast majority of the published literature focusing on its role in securing skin grafts [85–87]. Its efficacy as a primary treatment in the setting of acute burns, however, remains controversial [88]. A limited body of research has produced compelling evidence that thermal injuries managed acutely with NPWT demonstrate improved outcomes.

Morykwas et al*.* [89] were amongst the first to study the efficacy of NPWT in the acute management of burn injuries. In a porcine model, they created bilateral flank partial-thickness burns and applied NPWT to the burns on one side. Compared to the contralateral controls, burns treated with NPWT in the first 12 h following injury showed significantly reduced depth, cellular inflammation and collagen denaturation on histological analysis. The effect of NPWT on burn depth did not vary significantly with treatment duration. Applications of 5 days were no more efficacious than those spanning 12 or 6 hours.

The first human study to focus exclusively on NPWT for acute burns [90] likewise involved a comparison of differently treated bilateral thermal injuries. In seven patients presenting with bilateral partial-thickness hand burns, the more intensely injured hand underwent NPWT whilst the less injured hand received silver sulfadiazine. Daily measurements of wound perfusion in the first 3 days post-burn revealed a significant temporal decrease in blood flow in hands treated with silver sulfadiazine, but no such decrease in the hands given NPWT. Furthermore, the latter were significantly better perfused at days 2 and 3, and showed a clinically observable reduction in oedema formation. Healing times, however, were not reported. A prospective multicentre study with a similar design and a slightly larger sample size (*n* = 11) also found that the application of NPWT was associated with a greater reduction in oedema and increased perfusion compared to conventional silver-based therapy [91].

The remainder of the literature consists of a small number of case reports and retrospective reviews published over the past 13 years. Molnar et al. [49] described the case of a deep partial-thickness burn to the hand and forearm that fully re-epithelialised after 10 days following the application of NPWT. A comparison of the NPWT-treated burn with a less severe burn to the patient’s shoulder, which received standard therapy, revealed that NPWT yielded functionally and cosmetically superior results, with less hyperaemia and better skin quality. Another case report detailed the use of NPWT in a 54% TBSA full-thickness burn sustained by a civilian in the Iraqi war zone [92]. Although burns of this severity were typically fatal in Iraq, the patient survived his injury and was eventually discharged home. The authors concluded that the NPWT played a significant role in his survival.

The sole existing study to investigate the effect of NPWT on acute burns in children is a retrospective review by Ren et al. [93]. In a cohort of 29 paediatric patients, 22 of whom were undergoing treatment for burn injuries, the therapy was believed to accelerate wound granulation and decrease the number of required dressing changes, though the study did not include a control group and quantitative data on granulation tissue formation were not provided. Additionally, the authors failed to specify several salient details regarding their use of NPWT, including how long post-burn it was initially administered, whether it was given in combination with silver dressings, and the specific gauge pressure and duration of each NPWT application. Nevertheless, the lack of adverse events, with no recorded episodes of bleeding or abnormally elevated pain, attested to the safety of NPWT in the paediatric population.

Absent from the literature, however, are any high-powered trials with appropriate outcome measures comparing NPWT to standard acute burns management. The only published randomised controlled trial (RCT) to investigate the role of subatmospheric pressure in the management of acute burns was provided courtesy of Honnegowda et al*.* [94]. In their trial involving 50 adolescents and adults with thermal burns under 40% TBSA, half of the participants were allocated to intermittent NPWT and the other half to 5% povidone iodine gauze dressings. Wound biopsies collected at days 0 and 10 were subjected to a series of histological and biochemical analyses. The results revealed that the burns that underwent NPWT exhibited a richer, more stable extracellular matrix, less oxidative stress, an environmental pH more conducive to wound healing, and better overall granulation tissue deposition, angiogenesis and cellular infiltrate.

Although this trial shed significant light on the biochemical effects of NPWT, its focus on surrogate endpoints rather than validated patient-centred outcomes limited the clinical applicability of its findings. Additionally, the comparator control group was managed with betadine dressings not traditionally considered standard treatment in most developed countries. The study was further constrained by the time points of its data collection, which would fail to capture the full duration of re-epithelialisation for the majority of non-superficial burns.

The most recent Cochrane systematic review [95] on the application of NPWT in acute thermal injury, published in 2014, listed an earlier RCT that commenced in 2004. All available information on this trial is confined to a single conference abstract with interim data [96]. In the reported patient cohort, comprising a total of 23 adults presenting with bilateral partial-thickness hand burns, the NPWT-treated hands showed significantly reduced burn size at days 3 and 5 post-burn, but no difference at day 14. However, due to missing data (with a completed study never published in full) as well as several methodological limitations, the authors of the Cochrane review deemed the study to be at high risk of bias [95].

To address this gap in the literature, a pilot study was undertaken in 2015 at a paediatric burns outpatient department (OPD). In a sample of 20 children with acute burns, half were given a combination of NPWT and silver-impregnated dressings whilst the other half received silver dressings alone. Preliminary data revealed that the NPWT group exhibited moderately faster healing and reported lower pain scores [97]. More broadly, it demonstrated that NPWT could function as a feasible and safe addition to standard acute paediatric burns management and provided strong support for the development of a larger RCT.

## Methods/design

### Hypothesis and objectives

The central aim of this trial is to investigate the efficacy of NPWT as an adjunct to standard therapy in the treatment of paediatric burns. The research will measure the effects of NPWT on time to re-epithelialisation, pain and burn wound progression, and determine whether it produces outcomes superior to those of standard therapy alone. Based on data from a pilot study, it is hypothesised that NPWT will accelerate healing and reduce levels of pain and pruritus between dressing changes. Removal and re-application of the NPWT system is not expected to cause significantly more pain than standard dressing changes. In terms of the impacts on overall costs, it is suspected that NPWT may increase resource usage and costs in the acute phase of treatment, but, by virtue of faster re-epithelialisation and reduced scar formation, curb the personnel and material costs required for long-term scar management.

### Study design

**Fig. 1:**
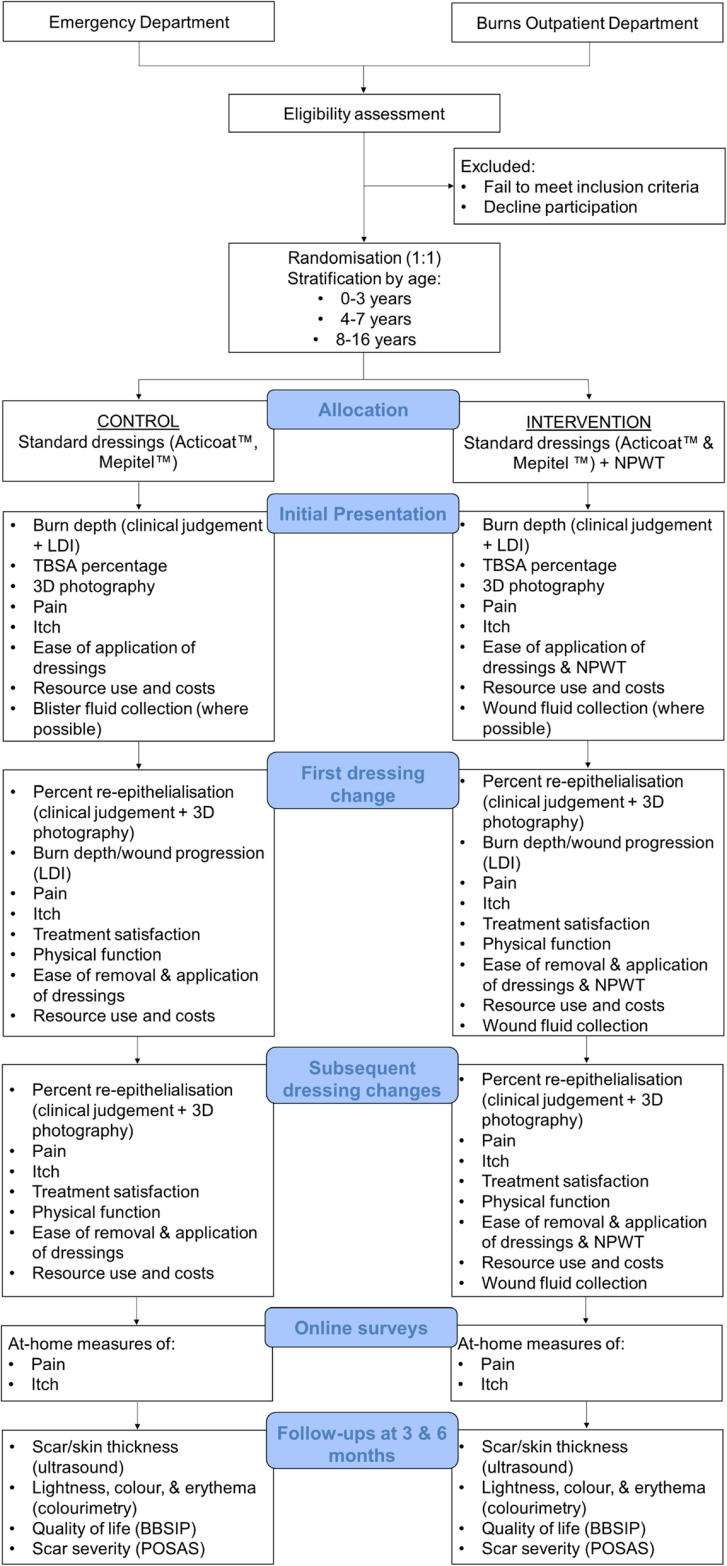
Study design flowchart. NPWT negative pressure wound therapy, LDI laser Doppler imaging, TBSA total body surface area, BBSIP Brisbane Burn Scar Impact Profile, POSAS Patient and Observer Scar Assessment Scale

**Fig. 2:**
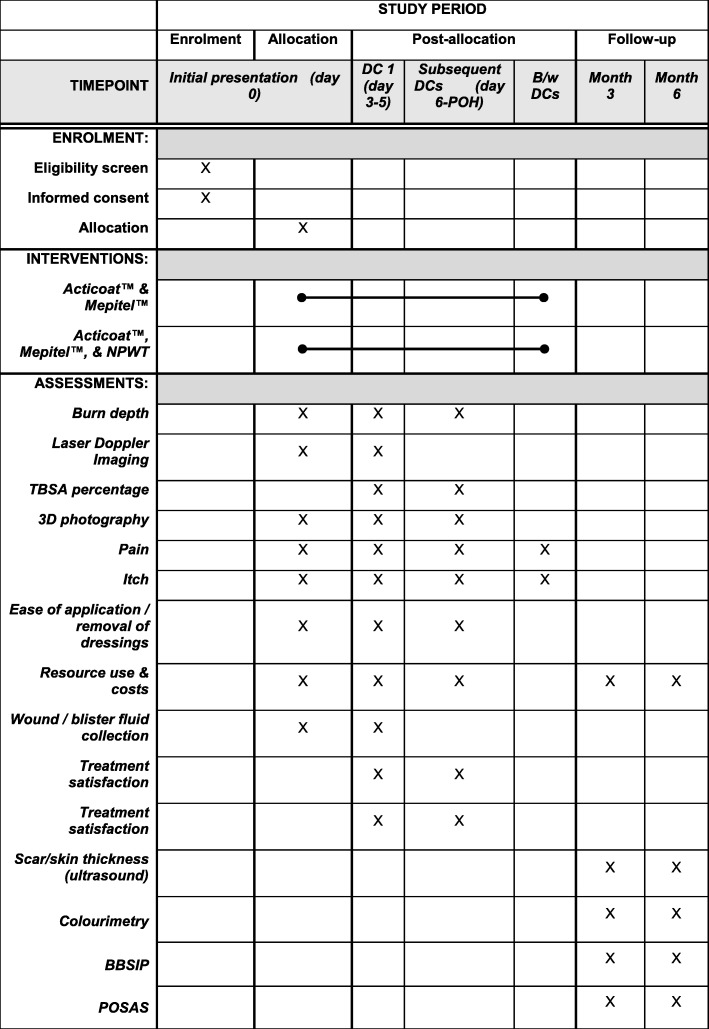
SPIRIT Figure. Schedule of enrolment, interventions, and assessments. B/w between, DC dressing change, POH point of healing, NPWT negative-pressure wound therapy, TBSA total body surface area, BBSIP Brisbane Burn Scar Impact Profile, POSAS Patient and Observer Scar Assessment Scale

This prospective RCT will be a superiority trial involving two parallel treatment arms: active control and intervention. Participants will be monitored throughout the course of the acute phase of their management, up until the point of healing, and then examined at 3- and 6-month follow-ups to assess the long-term outcomes of their burns. The different phases of the trial, and the measurements taken at each, are summarised in the study design flowchart (Fig. 1) and the Standard Protocol Items: Recommendations for Interventional Trials (SPIRIT) figure (Fig. 2). The SPIRIT checklist is provided in Additional file 1.

### Study setting

Patient recruitment will take place at a large metropolitan children’s hospital that serves as the sole provider of quaternary paediatric burns care for a region with a population of 4.5 million people.

#### Participants

##### Eligibility criteria

All paediatric burns patients presenting to the hospital’s emergency department (ED) or burns OPD will be considered for eligibility. Children will satisfy the inclusion criteria if they are under 17 years of age, possess a thermal burn covering <5% of their TBSA and present within 7 days of their injury. Exclusion criteria include burns that are located on the face or deemed by clinicians to be trivial in nature (i.e., not in need of further treatment). The eligibility criteria are listed in Table 1.

**Table 1:** Eligibility criteria

Inclusion criteria	Exclusion criteria
0–16 years of age TBSA < 5% Thermal burns Presentation to ED or burns OPD within 7 days of injury	Facial burns Trivial burns

*TBSA* total body surface area, *ED* emergency department, *OPD* outpatient department

##### Recruitment

All patients presenting to the ED or burns OPD with acute burns will be consecutively assessed for eligibility until the completion of recruitment. Nursing and surgical staff will identify eligible patients. In the ED, clinicians will first request permission from caregivers before inviting an investigator to approach them. If presenting to the burns OPD, potential participants will be approached only if they indicate a willingness to take part in research on an initial intake questionnaire.

An investigator will seek informed caregiver consent and child assent (for children 6 years and above) following the provision of verbal and written participant information. Once consent has been obtained, the patient will be randomised to one of the two treatment arms.

#### Interventions

Patients in the control group will receive the same standard dressings provided to all patients presenting to the burns OPD, which include a combination of Acticoat™ (Smith & Nephew, Hull, UK) and Mepitel™ (Mölnlycke Healthcare, Mikkeli, Finland), secured with Hypafix™ (BSN Medical, Hamburg, Germany). Likewise, the intervention group will first have their wounds dressed with Acticoat™ and Mepitel™. They will then additionally receive NPWT via the following protocol.

After applying the Acticoat™ and Mepitel™, clinicians will pack the burn with 10–20 layers of Kerlix™ (Medline Industries, Northfield, US). This antimicrobial gauze permits the vacuum to be distributed evenly across the entire wound site. In addition to their antimicrobial and moisturising properties, the silver-impregnated dressings will also serve as a wound contact layer, preventing encroachment of the Kerlix™ into the damaged tissue. An airtight seal will be generated via an adhesive film drape, to which tubing will be attached to connect the wound environment to a RENASYS TOUCH™ device (Smith & Nephew, Hull, UK). Suction will be set at a continuous subatmospheric pressure: 80 mmHg will be employed as the standard setting, but clinicians will be free to apply instead 40 mmHg in cases where ischaemia might be a concern (e.g., children under 1 year old with digit or extremity burns). Continuous pressures will be used in favour of intermittent cycles since past research showed that intermittent modes lead to elevated pain and the formation of undesirable granulation tissue [98]. The portability of the RENASYS TOUCH™ device, which can be carried around by children as young as 2 years of age in a custom-made pack, will allow patients to return to their everyday activities while still receiving NPWT.

All interventions will be carried out by burns clinicians with extensive experience in the application of dressings and NPWT. Following their initial presentation, participants will return to the OPD 3 to 5 days later for a change of dressings and, for those in the intervention group, a replacement of their NPWT system. To alleviate any additional pain from the removal of the adhesive drape, the clinicians replacing the NPWT apparatus will apply Niltac™ (ConvaTec, Greenlane, NZ), a silicone-based spray that can effectively dissolve the film and ease extraction. Participants will continue to present to the burns clinic every 3 to 5 days for dressing and NPWT changes until the wound is fully re-epithelialised or until grafting is required.

#### Randomisation

To accommodate the different scoring scales that will be used to assess pain, randomisation will be stratified by age into three groups: 0 to 3 years, 4 to 7 years, and 8 to 16 years. Allocation to the control arm and intervention arm of the study will occur at a 1:1 ratio. A random sequence will be generated by the trial statistician using the permuted block method with a random block size of either four or six participants. The statistician will then upload this sequence to the central randomisation module on the Research Electronic Data Capture application (REDCap; Vanderbilt University, Nashville, US). REDCap ensures allocation concealment by prohibiting investigators from viewing the sequence. Access is limited to an external administrator, who manages the server on which the module is hosted. Treatment allocations are assigned only after participants have been successfully enrolled and entered into the REDCap database. Randomisation will continue until each treatment arm has a minimum of 52 participants.

#### Blinding

The nature of the interventions precludes full blinding, but a number of key assessments will be blinded to minimise performance bias. By necessity, treating clinicians will be aware of group allocation throughout the duration of the participants’ care until the point of healing. Clinical judgements of re-epithelialisation, therefore, will not be blinded, but the use of 3D photographs will allow for a later review by a panel of blinded assessors. These assessors will not be provided any details pertaining to the participants’ management. If there any discrepancies, the blinded measures (or the majority thereof) will supersede those taken by clinicians.

Skin and scar assessments performed at 3- and 6-month follow-up appointments will also be blinded, as the clinical evaluators will have no knowledge of participants’ acute care. To reduce the risk of performance bias further, ultrasound images taken at these follow-ups will be reviewed by blinded assessors, who will measure scar and wound site thicknesses.

#### Primary outcome measure

##### Time to re-epithelialisation

The primary outcome will be time to full re-epithelialisation, as measured by the number of days from the injury to 95% re-epithelialisation of the burn wound. Percentage re-epithelialisation will be assessed at every clinical visit using multiple methods. A treating consultant will first perform an examination and record their assessment. Digital 3D photographs will then be taken of the burn using the 3DLife Viz II (Quantificare, Valbonne, France) and the D400 3D (Intel, Santa Clara, US) cameras. These photographs will be analysed using the software programs DermaPix (Quantificare, Valbonne, France) and 3D WoundCare (GPC, Swansea, UK), respectively, and undergo blinded review by a panel of three burns clinicians.

#### Secondary outcome measures

##### Pain

Pain intensity will be assessed at various time points throughout the participants’ management. At their first clinical visit, baseline pain measures will be recorded immediately prior to and following dressing applications. The same measures will be repeated before and after dressing changes at every subsequent clinical visit until the burn is considered fully re-epithelialised.

A variety of measures and age-dependent scales will be utilised to assess pain. Nurses will provide an observational rating using the Face, Legs, Activity, Cry, Consolability (FLACC) scale, which is accepted as a valid and reliable tool for assessing trauma-related pain behaviours in preverbal children [99, 100]. Children aged 4 to 7 years will be asked to self-report their pain using the Revised Faces Pain Scale (FPS-R) and those aged 8 years and older will self-report their pain using a numerical rating scale (NRS).

There is a large volume of literature demonstrating the validity and reliability of the FPS-R in children as young as 4 years old [101, 102]. For children aged 8 years and older, both the NRS and a visual analogue scale are well-validated tools [103, 104], but several comparison studies have expressed a preference for the NRS, which is associated with higher levels of responsivity, adherence and ease of use in the assessment of pain intensity among adults [105, 106]. Furthermore, the NRS allows for greater consistency throughout the full duration of the trial, as the two skin/scar assessment scales employed at the 3- and 6-month follow-ups both include numerical pain ratings.

To supplement the above pain ratings, investigators will additionally document the type and dosage of all analgesic medications administered to patients by health professionals or parents during the days on which they present to the clinic.

##### Pruritus

Itch severity will be measured concurrently with pain intensity. Unlike pain, emotional health, physical function and mobility, pruritus is one of the few domains in which parental and child perceptions significantly differ among paediatric burns patients [107]. As such, self-reports will be sought wherever possible. The only self-reporting itch scale that has been validated in children is the Itch Man Scale, which is recommended in children over the age of 5 years [108].

For patients too young to comprehend and cooperate with self-reporting scales, caregivers will be asked to perform an observational assessment using the Toronto Paediatric Itch Scale. This tool, which rates pruritus behaviours on a scale of 0 (absence of itch) to 3 (severe itch with significant disruption), has been found to assess itch severity in children aged 5 years or less with reasonable validity and reliability [109].

##### Pain and itch at home

Due to the multi-day durations of NPWT and silver-impregnated dressing treatments, measurements of pain and pruritus will be undertaken not only in the clinic but also at home, where most of the patients’ time undergoing their respective therapies will be spent. Caregivers will be contacted via text messages 24–48 h after every dressing application or change with a link to an online survey generated on REDCap. The survey will be automatically modified according to patient age to provide the appropriate pain and itch scales.

Retrospective reporting and recall bias are frequent risks associated with more traditional take-home questionnaires, with major implications for the validity of the outcomes they provide. [110–112] Online data collection via SMS notifications offers a valid alternative capable of prompting real-time assessments with a very high response rate and reduced management burden [113]. Moreover, any retrospective reports can be easily identified by their time stamps and evaluated accordingly.

If caregivers fail to complete the survey following the first message, they will be sent reminders every 24 h until they either submit their responses or attend their next clinical appointment. For those not in possession of a mobile phone or not agreeable to receiving research-related text messages, investigators will offer to send the surveys to their preferred email account, call their landline to collect the data verbally or, as a last resort, supply paper questionnaires.

##### Wound depth and progression

In addition to the treating consultant’s clinical assessments of burn depth and size, laser Doppler imaging (LDI) will be employed to help evaluate the extent of wound progression within burns. LDI measures blood perfusion, rather than directly assessing wound depth, but it is still widely considered to be a highly effective and accurate diagnostic tool for burn depth assessments [114]. As progression of wound depth and size is known to take place within the first 4 to 5 days post-burn [47, 115], LDI will be performed only at patients’ initial presentation and their first follow-up 3 to 5 days later. For each scan, investigators will record flux maximum, minimum, mean and standard deviation.

##### Treatment satisfaction

Clinicians in the burns OPD have observed anecdotally that older children and adolescents tend to be less tolerant of NPWT than younger patients. To evaluate quantitatively attitudes toward NPWT and how they vary with age, treatment satisfaction will be assessed at every clinical visit via the use of an 11-point NRS (0 = not at all satisfied; 10 = extremely satisfied). As with the NRS for pain, caregivers will provide an observational measure for children up to the age of 7 years, whilst those 8 years and older will self-report. Caregivers of all children will be asked to rate their own satisfaction with the treatment as well.

##### Physical function

Participants’ physical function while undergoing treatment will be assessed by patients and/or caregivers at every clinical visit, also via an 11-point NRS (0 = not at all easy to move; 10 = extremely easy to move).

##### Ease of management

The treating nurse will be surveyed on their views of the interventions after each baseline visit and change of dressings. They will specifically assess ease of removal and application, using another 11-point NRS (0 = not at all easy to remove/apply; 10 = extremely easy to remove/apply).

##### Scar and skin assessments

At 3 and 6 months following the burn injury, face-to-face follow-ups will be completed with all participants to conduct skin or scar reviews in conjunction with occupational therapy.

Ultrasound will be used to measure the thickness of the healed wound or scar. Scans will be taken centrally at each site of interest with the BT12 Venue 40 MSK ultrasound machine (General Electric, Little Chalfont, UK). The investigators will record and calculate an average of three thickness measurements within the central area at the time of the scan. These measurements will be compared to those taken from scans of healthy unburned skin contralateral to the site of the burn. Digital copies of the scans will be saved for later evaluations by blinded reviewers.

An objective quantification of the lightness, erythema, and pigmentation of the healed wound or scar will be obtained using the DSM II ColorMeter (Cortex Technology, Hadsund, Denmark). In paediatric studies, the device has exhibited high levels of inter-rater reliability [116], including among children with burn scars [117]. At each follow-up, two measurements will be taken of both the site of interest and a region of healthy skin for comparison.

The Patient and Observer Scar Assessment Scale (POSAS) will also be completed with the child (if over the age of 8 years) and caregiver. As a scar severity evaluation tool, the POSAS is both reliable and feasible [118–120], with an observer scale that shows adequate reliability in the assessment of scar appearance in children [117]. Another instrument, the Brisbane Burn Scar Impact Profile (BBSIP), will be used to measure scar patients’ physical and sensory symptoms as well as their health-related quality of life. Developed primarily for children, the BBSIP has undergone preliminary validation in paediatric patients at the participating burns centre [121].

##### Resource use and costs

Resource usage (and costs) for the care of each patient will be recorded from the perspective of the health service consistent with prior studies in the field [122, 123]. This will include details of the trial interventions, including the number of clinical visits, the duration of each visit, the dosages of analgesic medications prescribed, the quantity of dressings and NPWT products used, and associated labour time. Resource use will be costed at market rates (e.g., clinician time attributable to each participant will be costed based on relevant state-award salary rates). For patients requiring subsequent scar management, details of scar management (including scar interventions and associated clinician labour time) will also be recorded for each patient for the duration of the trial.

##### Grafting

With the inclusion of deep dermal partial-thickness and full-thickness burns in the eligibility criteria, it is anticipated that some participants will ultimately require skin grafting to achieve wound closure. All surgical procedures will be documented by investigators, but data collection will be discontinued for these patients at the point of grafting. Any data obtained prior to grafting will be included in the final analysis following an intention-to-treat methodology.

##### Adverse events

If a patient experiences an adverse event such as an infection or allergic reaction during the trial, investigators will record the event (even if not directly related to the child’s burn) and document any changes made to the child’s clinical care to address the issue. Should a consultant conclude that a particular dressing is not appropriate for a participant’s care, even in the absence of an adverse event, they will be free to select an alternative dressing. In both scenarios, data collection and analysis will adhere to intention-to-treat principles.

##### Wound exudate collection and analysis

Wound exudate has been increasingly recognised as a potentially rich source of information about the burn wound microenvironment [1]. Theoretically, NPWT is the ideal instrument for collecting this fluid. It extracts exudate directly from the interstitium and stores it in disposable canisters. Practically, however, the NPWT canisters have been engineered, for safety, to preclude any direct access to the collected exudate.

To obtain this fluid, therefore, investigators will inspect the used suction tube connected to the canister at every dressing change after the NPWT apparatus is replaced. If there is any visible wound aspirate, the tubing will be punctured so that the fluid can be drained into collection tubes. At the end of the clinical visit, the samples will be centrifuged at 855× relative centrifugal force for 5 minutes and frozen in aliquots at −80°C.

Once all the samples have been collected, they will be processed for SWATH™ mass spectrometry analysis, using techniques described previously [124]. Briefly, a 60-μg aliquot of each sample will be digested by trypsin, desalted, concentrated, and analysed using liquid chromatography-tandem mass spectrometry (LC-MS/MS) in data-independent acquisition mode for peptide identification and SWATH™ acquisition mode for peptide abundance. An existing peptide spectral ion library [125] will be used to identify the peptide products and generate proteomic profiles of the microenvironments of the different wounds. These profiles will be compared against one another as well as the proteomic profiles of paediatric burn blister fluid described previously by Zang et al. [125, 126]

The remainder of the samples will be subjected to more targeted assays to assess their levels of specific cytokines and other factors involved in the processes of burn wound conversion and healing. Enzyme-linked immunosorbent assay (ELISA) will be employed to detect markers of neovascularisation, re-epithelialisation and inflammation (e.g., VEGF, FGF-2, EGF, IL-8 and TNF-α). If sufficient quantities of exudate are collected, the assays will help to determine how these factors vary with time, depth and burn aetiology.

#### Sample size

The sample size was derived from the primary endpoint, time to re-epithelialisation. Previous burns research has reported a mean healing time with standard Acticoat™ therapy of 12.4 days (SD = 5.4) [127]. In light of findings by Deitch et al*.* [29] and Cubison et al*.* [30] that showed healing times under 10 days post-burn were associated with no hypertrophic scarring, the investigators considered that a 3.4-day reduction in time to re-epithelialisation would likely represent a clinically meaningful difference. Therefore, the study will aim to recruit a minimum of 104 participants for >80% power (assuming up to 20% drop-out and a significance level of 0.05). In 2017, approximately 1100 patients presented to the OPD for burns treatment. Of these, more than 90% satisfied the inclusion criteria of the present study. Therefore, it is expected that recruitment will be completed within a span of 9 months.

#### Data collection and management

Investigators will input data directly into the REDCap application via an electronic tablet. The application is hosted on a secure firewall-protected server at the research institute affiliated with the burns clinical unit. REDCap generates an audit trail that tracks all user activity, including data entry, manipulation and exportation. Any data exported for statistical analysis will be stored on a password-protected computer within the research institute. Access to passwords and codes will be limited to investigators involved in the trial. If any researcher leaves the project, all passwords will be replaced.

#### Data analysis

Conventional descriptive statistics will be used to describe the characteristics of the sample.

The potential effect of time to treatment, burn depth, burn TBSA, mechanism of injury, anatomical location of the burn and skin type will also be tested against primary and secondary measures in univariate analyses. Variables with *p* < 0.1 will be included as covariates in the primary and secondary analyses. Sensitivity analyses will also be conducted without adjustment for covariates.

Generalised linear (mixed) models will be prepared to examine primary and secondary outcomes across time and between groups, with patients as random effect where analyses include repeated measures within a patient. If mixed models do not converge with patient as a random effect, robust variance estimates for cluster-correlated data will be used [128]. For outcomes with repeated measures, the fixed effects of time, group and interaction time by group will be tested. Data will be analysed as intention-to-treat (primary analysis) and on a per protocol basis (sensitivity analysis) if deviations from the protocol occur. Missing data will be handled using multiple imputation where appropriate. Significance will be set at 0.05. Statistical analyses will be performed using Stata (StataCorp LLC College Station, TX:) or SPSS (IBM Corp, Armonk, NY, USA).

## Discussion

Over the past two decades, NPWT has been extensively applied and studied in the management of a wide variety of wounds, even prompting some authors to remark that it has “overwhelmed the wound-healing world” [129]. It is all the more surprising, then, that so little research has been conducted investigating the efficacy of the technique in the acute management of burns. In the limited volume of literature that does exist, there is promising evidence to suggest NPWT may reduce ischaemia, oedema formation and wound progression [49, 89–91]. However, the constraints of the animal model studies, case reports and retrospective studies that comprise the bulk of this literature cast substantial doubt on the applicability of their findings. To date, no results from appropriately powered RCTs with patient-centred outcomes have been published comparing NPWT to standard acute burns management [95, 130].

Furthermore, the underrepresentation of children in the NPWT literature (across all wound types, but in the context of acute burns particularly) overlooks a large segment of the burns patient population [5]. Children also tend to suffer disproportionately from some of the immediate and long-term risks that NPWT is believed to alleviate. It is believed that infants might be more sensitive to pain than adults [131], and the potential harms of hypertrophic scarring are far greater in patients still undergoing physical, social and psychological development [24].

Any attempts to extrapolate findings from the existing adult studies to a paediatric setting must be met with caution, not only due to the small sample sizes and methodological limitations of these trials, but also because young children differ markedly from adults in skin thickness and composition [132, 133], body surface area [134], and formation rates of granulation tissue [135]. The reluctance to employ NPWT in paediatric populations appears to stem from concerns about possible side effects, including bleeding, ischaemia and elevated pain [93]. However, reports of adverse events are rare in the literature, and the technique is widely regarded as safe when applied and monitored as per device instructions [136]. NPWT is known to cause pain at pressures greater than 125 mmHg [137], but this is well above the standard range preferred by the participating hospital. Anecdotally, the highest levels of pain experienced by paediatric patients undergoing NPWT are reported during removal of the adhesive film required to maintain the airtight seal. It is anticipated that with the application of Niltac™ prior to extraction, removing and re-applying the NPWT system will be no more painful than regular dressing changes. The overall risks of NPWT are, therefore, minimal, with most research focusing on the use of the therapy in children indicating that it is especially suited for paediatrics, as it reduces the frequency of procedures and allows for greater mobility than some standard dressings [138].

The trial was deliberately designed to serve as a pragmatic study of the efficacy of NPWT in the treatment of the broad spectrum of thermal burn injuries that routinely present to the participating ED and OPD. Especially for the latter, the burns seen over the course of even a single day tend to vary significantly in terms of first aid, prior treatment and time to presentation. For this reason, investigators have set a sample size that is large enough to account for the wide variability and allow for a statistical comparison of different groups.

The 7-day window for time to presentation was selected as it captures the majority of the OPD patient population. Although Morykwas et al*.* [89] found that NPWT was effective in their animal model only if administered within 12 h post-burn, there are strong reasons to believe that NPWT could continue to be of benefit to humans past this point. First and foremost are the pathophysiological differences between humans and animals, including the finding that the peak in post-burn oedema volume appears to occur later in humans than in non-human subjects [139–142]. Furthermore, multiple human studies have reported progression of burn surface area and depth over 4 to 5 days following injury [47], and high-protein oedema is known to remain in the interstitium for at least the first 7 days post-burn [139].

The range of assessments to be employed in this trial were selected for their reliability as well as the feasibility with which they could be carried out on paediatric patients. It is acknowledged that the age, size or distress of a patient may sometimes preclude researchers from completing certain assessments, such as 3D photography and LDI. To account for this potential limitation, additional measures of burn size, re-epithelialisation and depth will also be provided via the consultants’ clinical judgement.

### Study significance

Following a pilot study that yielded promising results, this trial aims to investigate the efficacy of NPWT in the treatment of acute paediatric burn injuries. By measuring the impact of NPWT on re-epithelialisation, burn wound progression and pain, the study aims to address the gaps in the NPWT literature and determine whether the therapy warrants implementation as an adjunct to standard therapy.

### Trial status

This manuscript represents the 15th version of the trial protocol, completed on 15 January 2019. Recruitment commenced on 2 May 2018 and is expected to conclude by 31 January 2019. At the time of writing, 103 participants have been recruited.

## Additional file

Additional file 1: SPIRIT Checklist. Recommended items to address in a clinical trial protocol (DOCX 49 kb)

## Abbreviations

BBSIP: Brisbane Burn Scar Impact Profile
B/w: Between
DC: Dressing change
ED: Emergency department
FLACC: Face, Legs, Activity, Cry, Consolability
FPS-R: Faces Pain Scale, Revised
LDI: Laser Doppler imaging
NPWT: Negative pressure wound therapy
NRS: Numerical rating scale
OPD: Outpatient department
POH: Point of healing
POSAS: Patient and Observer Scar Assessment Scale
RCT: Randomised controlled trial
SMS: Short Message Service
SWATH: Sequential window acquisition of all theoretical fragment-ion spectra
TBSA: Total body surface area
